# Two Pairs of *Drosophila* Central Brain Neurons Mediate Larval Navigational Strategies Based on Temporal Light Information Processing

**DOI:** 10.3389/fnbeh.2018.00305

**Published:** 2018-12-05

**Authors:** Tim-Henning Humberg, Simon G. Sprecher

**Affiliations:** Department of Biology, Institute of Zoology, University of Fribourg, Fribourg, Switzerland

**Keywords:** *Drosophila* larvae, NP394, insect behavior, light navigation, temporal information processing, spatial information integration, behavioral plasticity, visual system

## Abstract

Some animals are attracted by sun light, others are highly repulsed by it. Especially for slowly moving animals, such as *Drosophila* larvae, direct sunlight may be perceived as noxious stimulus as it increases the risk of desiccation, DNA-damaging by UV-light and exposure to predators. For several reasons, model organisms like *Drosophila* larvae are well-suited for investigating how light cues are translated into an appropriate behavioral output. First, many of the genetic tools, which were created for use in adult fruit flies, work also in larvae. Second, the lower number of cells in *Drosophila* larvae compared to adults makes this system adequate for reconstructing neural circuits. Third, the relatively simple behavioral repertoire of larvae facilitates the study of basic functions like navigation with regards to light. Larvae navigate robustly away from a light source by the use of several sophisticated behavioral strategies which are based on temporal or spatial information processing. Two central brain neurons, the NP394-neurons, are highly important for larval light avoidance. It was even reported that these cells seem to play a functional role in a putative larval light preference switch right before pupation. However, the exact function of the NP394-neurons in light navigation remains unknown. We here show that the functional role of NP394-neurons in larval light navigation is restricted to behaviors based on temporal information processing, but not for spatial navigation.

## Introduction

*Drosophila* larvae are highly photophobic and navigate robustly away from a light exposure by using sophisticated behavioral strategies either based on temporal or spatial information processing (Kane et al., 2013; Humberg and Sprecher, 2017; Humberg et al., 2018).

One navigational strategy based on temporal information integration is biasing the head sweep acceptance rate (Kane et al., 2013; Humberg et al., 2018). Larvae terminate their runs to probe their environment via head sweeping. A rejected head sweep is followed by another head sweep to the opposite direction. An accepted head sweep is followed by a turn toward this direction. Larvae are more likely to reject a head sweep when sensing light intensity increase and they are more likely to accept a head sweep when sensing a decrease in light intensity (Kane et al., 2013; Humberg et al., 2018). Further, larvae are more likely to initiate a turn and perform a turn of greater size when sensing light intensity increase and not decrease (Kane et al., 2013; Humberg and Sprecher, 2017; Humberg et al., 2018).

Beside temporal changes in light intensity, larvae perceive and process also spatial light intensity differences (Humberg et al., 2018). By comparing the input of their two eyes, larvae steer within a run away from a light source or bias the direction of their first head sweep away from the light stimulus (Humberg et al., 2018). Thus, the final turn direction is a composition of head sweep direction and acceptance rate (Kane et al., 2013; Humberg et al., 2018).

**Figure 1:**
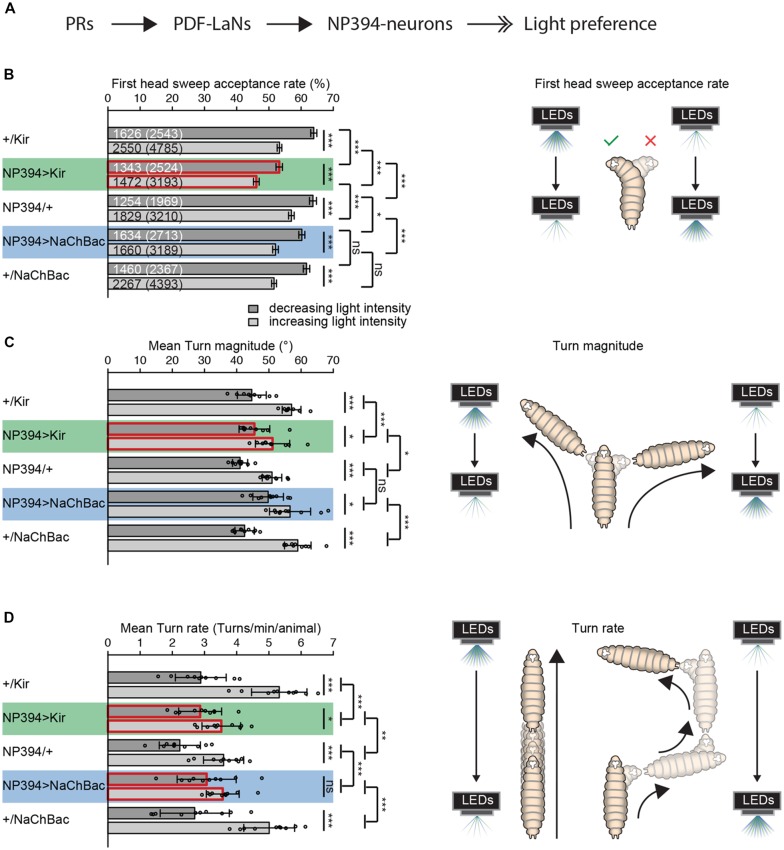
The functional role of NP394-neurons in navigational strategies based on temporal information processing.** (A)** Flow chart is illustrating the known upstream partners of NP394-neurons, namely photoreceptor neurons (PRs) and *Pigment dispersing factor* expressing lateral neurons (PDF-LaNs). NP394-neurons play a role in larval light preference (solid black arrows with open double-heads). **(B–D)** Either Kir2.1 or NaChBac were expressed in NP394-neurons to silence or hyper-activate the cellular activity of these neurons, respectively. Larvae were tested in the temporal light gradient assay (please see “Materials and Methods” section). **(B)** Functional NP394-neurons are not necessary for accepting more head sweeps during a decrease than increase of light intensity. Silencing neuronal activity of NP394-neurons leads to a decreased first head sweep acceptance rate in comparison with the corresponding controls for light intensity in- and decrease respectively. **(C)** Functional NP394-neurons are not necessary for making turns of greater size during an increase than decrease of light intensity. Animals with silenced NP394-neurons activity possess defects in biasing their turn size with respect to the corresponding controls. **(D)** Hyper-activation of NP394-neurons cellular activity seems to lead to a loss of the turn rate bias. Larvae with silenced NP394-neurons cellular activity show a decreased turn rate bias compared to the respective controls. **(A–C)** *p < 0.05, **p < 0.01, ***p < 0.001, ns, not significant. Data show mean. **(B)** First numbers are number of accepted first head sweeps. Numbers in brackets are total number of first head sweeps. Error bars show SEM. **(C,D)** Circles indicate means of individual experiments and error bars show SD. Please find information about the used statistical tests and the exact results of statistical analysis in Supplementary Table S1.

Larval eyes are essential for phototaxis (Kane et al., 2013; Humberg and Sprecher, 2017; de Andres-Bragado et al., 2018; Humberg et al., 2018). Photoreceptor neurons (PRs) of the larval eyes synapse on different visual interneurons like the four *Pigment dispersing factor* expressing lateral neurons (PDF-LaNs; Larderet et al., 2017; Figure 1A). PDF-LaNs are upstream of two pairs of prothoracicotropic hormone (PTTH) expressing central brain neurons called NP394-neurons (Gong et al., 2010; Yamanaka et al., 2013; Figure 1A). Light-dark choice tests revealed that these NP394-neurons are highly essential for larval light avoidance and serve as a neuronal substrate of behavioral plasticity by mediating a switch in larval light preference (Gong et al., 2010; Yamanaka et al., 2013). However, these preference tests lack detailed information about which behavioral strategy are affected in case NP394-neurons are not properly functioning.

We here address the functional role of NP394-neurons in different navigational strategies. By combining a tracking system with temporal or spatial light stimulation as well as with genetically interference of NP394-neurons cellular activity, we show that NP394-neurons seem to play a role in navigational strategies based on temporal light information processing, but seem to be dispensable for strategies based on spatial light information processing.

## Materials and Methods

### Animals

*Drosophila melanogaster* were raised at 25°C in a 12:12 light-dark cycle on cornmeal medium. Thirty foraging third instar larvae were used for each experiment. Lines used: w*;NP394-Gal4/TM6b (Kyoto-Stock #103604), w*;UAS-Kir2.1 (Bloomington-Stock #6596), yw*;UAS-NaChBac (Bloomington-Stock #9467).

### Preparation of Experiments

Experiments were performed during the animals’ subjective day. Larvae were in darkness at least 20 min before an experiment. Larvae were collected in water droplets for up to 10 min before an experiment. The testing plate was a 24.5 × 24.5 cm petri dish containing 2% agarose (Agarose Standard, Roth).

### Tracking System

Experiments were performed in a box with red LEDs illumination (623 nm). Larvae acclimatized to the plate for 1 min. For 10 min larval behavior was recorded with 13 frames/s by a camera (acA2500-14gm, Basler AG) equipped with a Fujinon lens (Fujinon HF12.5HA-1B 12.5 mm/1.4, Fujifilm) and a light red bandpass filter (BP635, Midwest Optical Systems). Customized softwares were used for image acquisition and data analysis (Gershow et al., 2012; Kane et al., 2013; Humberg et al., 2018).

### Visual Stimulation

Larvae were stimulated with two different lighting schemes also used in a recent study (Humberg et al., 2018).

Directional light source assay: a projector (EX7200 Multimedia Projector, EPSON) equipped with a bandpass filter (335–610 nm, BG40, Thorlabs) was located 26 cm in height and 38 cm in distance away from the plate center and orientated 40°. Maximum light intensity was 4,331 μW/cm^2^. We measured two maximum intensity peaks. The first one was at 438 nm (71.6 μW/cm^2^) with half widths of 9 nm and the second one was at 594 nm (47.9 μW/cm^2^) with half widths of 10 nm.

Temporal light source assay: a blue and a green LED (PT-120, Luminus, Billerica) were used together and were located 45 cm in height above the plate. The highest intensity was 378 μW/cm^2^. One maximum intensity peak was at 455 nm (11.9 μW/cm^2^) with half widths of 9 nm. A second maximum intensity peak was at 522 nm (3.7 μW/cm^2^) with half widths of 14 nm. The LEDs intensity was changing every 100 ms by 1.5 μW/cm^2^ driven by an Arduino running a custom-made code (Humberg et al., 2018). This lighting scheme possess mainly two distinct phases of either linear light intensity increase or decrease, respectively.

These two types of phases were lasting both for 25.5 s and were spaced by 4.5 s of constant high or low light intensity, respectively. The phases of constant light intensity ±1 s were not considered for analysis.

### Navigational Parameters

Behavioral parameters were defined and analyzed as described previously (Humberg et al., 2018). Briefly, runs are events of forward locomotion with larval head and body aligned. Turns are events of no forward locomotion with larval head and body not aligned (head sweeping). A turn consists of at least one head sweep. Rejected head sweeps are followed by another head sweep. An accepted head sweep terminates the turn and initiates a run in this new direction.

Data from the directional light source assay were analyzed as followed. We used a navigational compass: larval heading toward the light source (0°); away from the light source (180°) and perpendicular to the light source (+90° or −90°). The compass directions were split into four bins of each 90° in size.

Turn direction: only turns that followed a previous heading direction of ±90° were analyzed. It was analyzed if heading direction after the turn was orientated more toward or away from the light source in comparison to previous heading direction.

Steering within runs: only runs that followed a previous heading direction of ±90° were analyzed. The difference in heading direction between the start and the end of each run was calculated.

First head sweep direction: only first head sweeps were analyzed. It was determined if the direction of a first head sweep was orientated toward or away from the light source in comparison to previous heading direction.

Data from the temporal light source assay were analyzed as followed: first head sweeps and turns were analyzed separately for the phases of light intensity increase and decrease, respectively.

First head sweep acceptance rate: the probability that first head sweeps got rather accepted than rejected was calculated.

Turn size: the turn size was the difference between the previous heading direction and the heading direction after the turn. A mean turn size was determined for each experiment. An overall mean was calculated from these individual means.

Turn rate: all turns initiated during a certain intensity change phase (23.5 s) were counted (x turns). These numbers were converted into turns/min by dividing this number by 23.5 s and multiplying the result with 60 s ((x turns/23.5 s) * 60 s). These numbers were divided by the average larvae number on the plate during the corresponding phase. Thus, for each experiment 10 means were determined for both the phases of intensity in-and decrease, respectively. From these means of single phases of individual experiments a mean was determined for each experiment. An overall mean was determined from these means of single experiments.

## Results

### Navigational Strategies Based on Temporal Light Information

First, we investigated if NP394-neurons possess a functional role in mediating navigational strategies based on temporal information processing. We genetically silenced NP394-neurons by expressing a potassium channel Kir2.1 or genetically hyper-activated these neurons by expressing the bacterial sodium channel NaChBac (Baines et al., 2001; Nitabach et al., 2006; Pauls et al., 2015).

Larvae with silenced NP394-neurons are still able to reject more head sweeps when they sense a light intensity increase and accept more head sweeps when they sense an intensity decrease (Figure 1B). However, the head sweep acceptance rates for both phases of light intensity in- and decrease are lower compared to the data from the respective controls (Figure 1B). Larvae in which NP394-neurons were hyper-activated possess lower first head sweep acceptance rates only compared to their driver line control (Figure 1B).

Larvae with not properly functioning NP394-neurons bias their mean turn size (Figure 1C). However, this bias is less prominent in comparison with the bias of corresponding controls (Figure 1C). The turn size biases of larvae in which NP394-neurons were hyper-activated does not differ from control animals (Figure 1C).

Animals with silenced NP394-neurons turn more often when they sense an increase in light intensity (Figure 1D). However, the turn rate bias is much lower compared to the one of the corresponding controls (Figure 1D). Animals in which NP394-neurons were hyper-activated do not seem to bias their turn rate (Figure 1D).

Thus, NP394-neurons seem to be involved in driving navigational strategies based on temporal light intensity information.

### Navigational Strategies Based on Spatial Light Information

**Figure 2:**
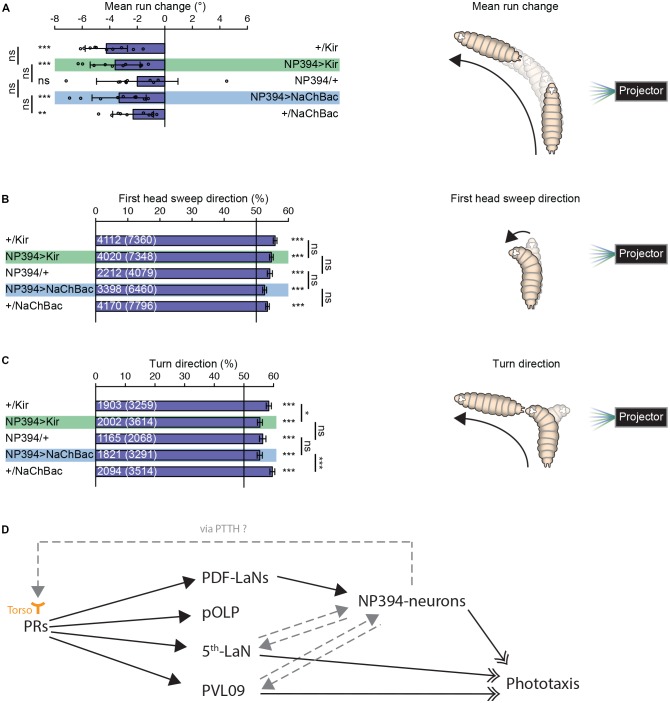
Navigational strategies based on spatial information processing.** (A–C)** Larvae were tested in the spatial light source assay (please see “Materials and Methods” section). **(A)** Larvae with silenced or hyper-activated NP394-neurons activity are able to steer away from a light source. Larvae with silenced or over-activated NP394-neurons activity showed not a decreased steering with in runs away from the light source compared to both of the corresponding control lines. **(B)** These larvae are able to bias already the first head sweep away from the light source. Moreover, the probability of the first head sweep direction of larvae lacking functional NP394-neurons is not statistically different from the respective control lines. **(C)** Further, these animals are able to bias their final turn direction away from the light source. Moreover, the probability of the turn direction of larvae lacking functional NP394-neurons is only statistically different from the respective effector line control. **(A–C)** *p < 0.05, **p < 0.01, ***p < 0.001, ns, not significant. Data show mean. **(A)** Circles indicate means of individual experiments and error bars show SD. **(B,C)** First numbers are number of first head sweeps or turns directed away from the light source, respectively. Numbers in brackets are total number of first head sweeps or turns, respectively. Error bars show SEM. Please find information about the used statistical tests and the exact results of statistical analysis in Supplementary Table S1. **(D)** Flow chart is illustrating the known (solid black arrows with closed head) and putative (non-solid gray arrows with closed head) upstream and downstream partners of NP394-neurons, namely PRs, PDF-LaNs, projection optic lobe pioneer cell (pOLP), postero-ventro-lateral neuron 09 (PVL09) and the 5th-LaN. NP394-neurons could feedback on PRs via prothoracicotropic hormone (PTTH) which could bind to Torso of PRs. NP394-neurons, PVL09 and the 5th-LaN play a role in phototaxis (solid black arrows with open double-heads).

Properly functioning NP394-neurons are not necessary for larvae to steer within runs away from a light source (Figure 2A). Larvae with silenced or hyper-activated NP394-neurons bias their first head sweep direction away from the light source (Figure 2B). Further, all groups bias their turn direction away from the light source (Figure 2C). However, larvae with silenced and hyper-activated NP394-neurons possess a turn direction bias which is lower compared with the respective effector line controls (Figure 2C).

Taken together, NP394-neurons seem to be dispensable for mediating navigational strategies based on spatial light intensity information.

## Discussion

### The Functional Role of NP394-Neurons in Visually-Driven Behavior

Previous studies showed that NP394-neurons are absolutely essential for larval light avoidance by using light-dark choice tests (Gong et al., 2010; Yamanaka et al., 2013). These tests consist of homogenously illuminated and shaded areas. Thus, larvae might only sense light intensity differences when moving between the two different area types. Within these tests, larval behavioral decisions might be based on temporal intensity changes. Thus, in line with our results, NP394-neurons seem to function in mediating navigational strategies based on temporal light information processing. Further, our data suggests that the function of NP394-neurons could be restricted solely to behavioral strategies based on temporal light information integration.

Our results do not confirm a functional role of NP394-neurons in a light preference switch. Further, in our experiments larvae with hyper-activated NP394-neurons do not show increased light avoidance (Gong et al., 2010). Our data suggests that genetically silencing and hyper-activating NP394-neurons activity lead rather to similar than opposite effects on larval performance of navigational strategies. We speculate that light increment and decrement could lead respectively to opposite effects on NP394-neurons’ neuronal activity (increase vs. decrease of action potential frequency). Thus, information about temporal light cues would be transmitted by temporal changes of neuronal activity. It seems likely that either chronically silencing or hyper-activating NP394-neurons would interfere with the proper transmission of temporal variations in cell activity. In turn, this inability would result in similar defects on larval performance of navigational strategies based on temporal information processing. That genetically silencing as well as hyper-activating the same neurons activity can lead to similar defects in visually-driven behavior is also reported elsewhere (Pauls et al., 2015). However, the use of different assays, analysis and effector lines can lead to different results. All three studies have in common that NP394-neurons are highly important for larval light behavior (Gong et al., 2010; Yamanaka et al., 2013).

### The Information Flow From the Visual System to NP394-Neurons … and Back?

The exact position of NP394-neurons within the larval visual system is unknown. It was shown that NP394-neurons are downstream of PDF-LaNs (Gong et al., 2010; Figure 2D). NP394-neurons respond faster and stronger to light stimulation in absence of PDF-LaNs (Gong et al., 2010). Thus, PDF-LaNs seem to only modulate NP394-neurons and NP394-neurons might receive light information also independently from PDF-LaNs via other first-order visual projection neurons. Other first-order visual projection neurons are projection optic lobe pioneer cell (pOLP), postero-ventro-lateral neuron 09 (PVL09) and the 5th-LaN (Larderet et al., 2017; Figure 2D). No functional role of PDF-LaNs and pOLP in phototaxis is described (Humberg et al., 2018). However, silencing all LaNs or PVL09 lead to defects in navigational strategies based on both temporal and spatial information processing (Humberg et al., 2018). Therefore, the 5th LaN and/or PVL09 could be upstream of NP394-neurons (Figure 2D). How these projection neurons transmit only temporal light information to NP394-neurons remains to be investigated. Otherwise, NP394-neurons use PTTH to control light avoidance (Yamanaka et al., 2013). PTTH activates Torso and knockdown of *torso* in PRs lead to loss of light avoidance (Yamanaka et al., 2013). Thus, the function of NP394-neurons for phototaxis could be indirect by feeding back on PRs and/or visual projection neurons (Figure 2D). Now, future anatomical studies on the larval visual connectome might reveal the connectivity between the NP394-neurons and all their up- and downstream partners. This neuronal roadmap will guide physiological studies which might reveal how all these cells respond to stimulation with temporal and spatial light stimuli.

## Data Availability

The raw data supporting the conclusions of this manuscript will be made available by the authors, without undue reservation, to any qualified researcher.

## Author Contributions

T-HH and SS designed the experiments and wrote the manuscript. T-HH performed the experiments, analyzed the data and created the figures.

## Conflict of Interest Statement

The authors declare that the research was conducted in the absence of any commercial or financial relationships that could be construed as a potential conflict of interest.

## Supplementary Material

TABLE S1 Information about the used statistical tests and results of statistical analysis. Standard statistic functions in MatLab (“*t*-test”) and RStudio (“binom.test,” “fisher.test,” “aov” and “glht (multcomp)”) were used for statistical analysis. In case of multiple testing, *p*-values were adjusted according to the Benjamini Hochberg procedure.

## References

[B1] Baines R. A. Uhler J. P. Thompson A. Sweeney S. T. Bate M. (2001). Altered electrical properties in *Drosophila* neurons developing without synaptic transmission. J. Neurosci. 21, 1523–1531. 10.1523/jneurosci.21-05-01523.2001 11222642 PMC6762927

[B2] de Andres-Bragado L. Mazza C. Senn W. Sprecher S. G. (2018). Statistical modelling of navigational decisions based on intensity versus directionality in *Drosophila* larval phototaxis. Sci. Rep. 8:11272. 10.1038/s41598-018-29533-0 30050066 PMC6062584

[B3] Gershow M. Berck M. Mathew D. Luo L. Kane E. A. Carlson J. R.. (2012). Controlling airborne cues to study small animal navigation. Nat. Methods 9, 290–296. 10.1038/nmeth.1853 22245808 PMC3513333

[B4] Gong Z. Liu J. Guo C. Zhou Y. Teng Y. Liu L. (2010). Two pairs of neurons in the central brain control *Drosophila* innate light preference. Science 330, 499–502. 10.1126/science.1195993 20966250

[B6] Humberg T. H. Bruegger P. Afonso B. Zlatic M. Truman J. W. Gershow M.. (2018). Dedicated photoreceptor pathways in *Drosophila* larvae mediate navigation by processing either spatial or temporal cues. Nat. Commun. 9:1260. 10.1038/s41467-018-03520-5 29593252 PMC5871836

[B5] Humberg T. H. Sprecher S. G. (2017). Age- and wavelength-dependency of *Drosophila* larval phototaxis and behavioral responses to natural lighting conditions. Front. Behav. Neurosci. 11:66. 10.3389/fnbeh.2017.00066 28473759 PMC5397426

[B7] Kane E. A. Gershow M. Afonso B. Larderet I. Klein M. Carter A. R.. (2013). Sensorimotor structure of *Drosophila* larva phototaxis. Proc. Natl. Acad. Sci. U S A 110, E3868–3877. 10.1073/pnas.1215295110 24043822 PMC3791751

[B8] Larderet I. Fritsch P. M. J. Gendre N. Neagu-Maier G. L. Fetter R. D. Schneider-Mizell C. M. (2017). Organization of the *Drosophila* larval visual circuit. Elife 6:e28387 10.7554/elife.28387 PMC5577918 30726702

[B9] Nitabach M. N. Wu Y. Sheeba V. Lemon W. C. Strumbos J. Zelensky P. K.. (2006). Electrical hyperexcitation of lateral ventral pacemaker neurons desynchronizes downstream circadian oscillators in the fly circadian circuit and induces multiple behavioral periods. J. Neurosci. 26, 479–489. 10.1523/jneurosci.3915-05.2006 16407545 PMC2597197

[B10] Pauls D. von Essen A. Lyutova R. van Giesen L. Rosner R. Wegener C.. (2015). Potency of transgenic effectors for neurogenetic manipulation in *Drosophila* larvae. Genetics 199, 25–37. 10.1534/genetics.114.172023 25359929 PMC4286689

[B11] Yamanaka N. Romero N. M. Martin F. A. Rewitz K. F. Sun M. O’Connor M. B.. (2013). Neuroendocrine control of *Drosophila* larval light preference. Science 341, 1113–1116. 10.1126/science.1241210 24009394 PMC3906047

